# The Effect of Intravenous Parecoxib on Early Ambulation After Elective Lumbar Spinal Surgery: A Propensity Score Matching Analysis

**DOI:** 10.3390/jcm14228005

**Published:** 2025-11-11

**Authors:** Busakorn Ruksouy, Tanyong Pipanmekaporn, Pathomporn Pin-on, Pichitchai Atthakomol, Piyada Boonsong

**Affiliations:** 1 Department of Anesthesiology, Phichit Provincial Hospital, Phichit 66000, Thailand; rugsouy1540@gmail.com; 2 Department of Anesthesiology, Faculty of Medicine, Chiang Mai University, Chiang Mai 50200, Thailand; tanyong24@gmail.com (T.P.); piyadaster@gmail.com (P.B.); 3 Department of Biomedical informatics and Clinical Epidemiology, Faculty of Medicine, Chiang Mai University, Chiang Mai 50200, Thailand; p.atthakomol@gmail.com; 4 Department of Orthopaedics, Faculty of Medicine, Chiang Mai University, Chiang Mai 50200, Thailand

**Keywords:** postoperative pain control, early ambulation, lumbar spine surgery, parecoxib

## Abstract

**Background:** Intravenous (IV) parecoxib could reduce pain intensity during the acute phase of lumbar spine surgery. However, details pertinent to the specific effect of this medication on promoting early patient mobility are still controversial. We aimed to investigate the effect of intravenous parecoxib on promoting early ambulation. **Methods:** This retrospective observational study included patients who underwent elective lumbar spinal fusion between January 2017 and December 2021. The electronic medical records were reviewed, and the patients were divided into two groups: those who received intravenous parecoxib and those who received routine opioid therapy. Propensity score matching (PSM) was employed. The incidence of early ambulation within 24 h after surgery. Patients in both groups were compared. **Results:** A total of 397 patients’ medical records were reviewed. After one-to-one PSM, there were 125 patients in each group. The incidence of early ambulation was higher in the parecoxib group (56% vs. 40%, *p* = 0.020). The average time point at which patients could ambulate was 10.4 h earlier in the parecoxib group compared to the control group (95% CI −12.84 to −8.06, *p* < 0.001). **Conclusions:** The administration of intravenous parecoxib after lumbar spine surgery demonstrated encouraging effects on early ambulation, with patients able to get out of bed and walk within 24 h after surgery. To endorse intravenous parecoxib as a standard postoperative pain protocol for spine surgery, further investigation in randomized controlled trials should be conducted.

## 1. Introduction

Extreme pain following spine fusion procedures is common, particularly during the first 72 h [1]. Insufficient pain management has been shown to result in serious consequences such as deep vein thrombosis (DVT), pulmonary embolism (PE), systemic infection, and prolonged hospitalization [2,3,4,5]. Providing immediate relief for patients experiencing severe pain after spine surgery is essential as it enhances overall surgical results, increases patient satisfaction, decreases hospital stays, and improves cost efficiency [6,7]. Intravenous opioids are commonly prescribed for postoperative pain control, guided by clinical indications and the patient’s pain intensity [8]. The unpleasant side effects of opioids include intestinal ileus, nausea, vomiting, pruritus, respiratory depression, depression of the central nervous system, and urine retention [9,10].

The modern tendency for enhancing recovery from anesthesia has rendered the sole use of opioid analgesics obsolete. The Enhanced Recovery After Surgery (ERAS) program has advocated the opioid-sparing approach to encourage early recovery and reduce the adverse effects of opioids. Multimodal opioid-sparing methods included acetaminophen, gabapentin, alpha-2-agonists, ketamine, magnesium sulfate, high-dose steroids, local anesthetics, ketorolac, and nonsteroidal anti-inflammatory drugs (NSAIDs) [2,3,4,10,11]. Cyclooxygenase 2 (COX-2) specific inhibitors are strongly recommended for pain management following lumbar laminectomy, as cited in the procedure-specific postoperative pain management (PROSPECT) guidelines [2,3].

Research has been undertaken to evaluate the efficacy of parecoxib, a selective COX-2 inhibitor often prescribed for acute pain following spine surgery. This research has explored different administration regimens, which vary in their timing and sequence. These factors may influence the quality of pain control and, consequently, the time to early ambulation [12,13]. Low to moderate supportive evidence was found for the use of parecoxib in a recent meta-analysis of twenty-seven trials (*n* = 2840) comparing intravenous parecoxib with placebo or other active medications for acute postoperative pain in patients following spine surgery, demonstrating significant reductions in pain scores and opioid consumption without an associated increase in adverse events [14,15]. A non-randomized controlled study demonstrated that intravenous parecoxib significantly decreased pain severity and enhanced functional independence (as measured by the Barthel index) when compared to patient-controlled analgesia [12].

The effect of parecoxib on the time to early ambulation, postoperative complications, and length of hospital stay compared to conventional opioid analgesia is not well established. This study aims to evaluate the efficacy of intravenous parecoxib can shorten the time to early ambulation after lumbar spine surgery compared to conventional opioid analgesia using a propensity score matching approach.

## 2. Material and Methods

### 2.1. Study Design and Patient Cohort

A retrospective cohort study was conducted from 2017 to 2021, recruiting patients who underwent elective lumbar spine surgery. The diagnoses according to the International Classification of Diseases 10 (ICD-10) were spinal canal stenosis, spondylosis, and spondylolisthesis of the lumbar level. The inclusion criteria included patients aged 18 years who underwent elective lumbar spine surgery at Phichit Provincial Hospital. Participants were excluded if they had a history of previous spine injuries, spinal malignancies, failed back surgery, or chronic opioid use. Other exclusion criteria included undergoing minimally invasive surgery, dependence on mechanical ventilation, lower motor weakness, death within 24 h of surgery, or transfer to another hospital.

The study protocol was approved by the Institutional Review Board (IRB) of Phichit Provincial Hospital (protocol number: 0194/2022). The medical records were reviewed by a nurse anesthetist. Patient characteristics, including age, gender, and body mass index (BMI), were collected, and any comorbidities (coronary artery disease: CAD, chronic obstructive pulmonary disease: COPD, diabetes, chronic kidney disease: CKD) were recorded. The surgical details, including the type of spinal fusion, the number of fusion levels, and the duration of surgery, were noted. Time to early ambulation was defined as the duration in hours from the completion of surgery until the patient was able to get out of bed and commence walking, either independently or with assistance. Pain levels at rest and during movement were evaluated using a numeric rating scale (NRS) at 2, 6, 12, 24, 48, 72, and 96 h postoperatively. Degree of pain severity was categorized as mild, moderate, or severe. The intensity of pain was rated on a scale from 0 to 10, where 0 indicated no pain and 10 represented the worst imaginable pain. Pain rated as moderate was defined as an NRS score of 4–6, while severe pain corresponded to a score of 7–10 [16,17]. The total amount of opioids administered both intraoperatively and in the 96 h following surgery was also recorded. Adverse events related to parecoxib, including gastrointestinal (GI) side effects, rash, and Acute Kidney Injury (AKI). AKI was defined according to the Kidney Disease: Improving Global Outcomes (KDIGO) criteria as an increase in serum creatinine of ≥0.3 mg/dL within 48 hours or an increase to ≥1.5 times the baseline value. [18]. Length of hospital stay (LOS) was defined as the total number of days from the date of hospital admission to the date of discharge.

### 2.2. Anesthesia, Surgery, and Perioperative Care

In the operating theater, standard intraoperative monitoring, including non-invasive blood pressure, three-lead electrocardiogram (ECG), heart rate, pulse oximetry, end-tidal carbon dioxide (ETCO_2_), oropharyngeal temperature, and urine output, was applied to all patients. General anesthesia was induced with intravenous propofol (1–2 mg/kg) and facilitated by a non-depolarizing muscle relaxant, cisatracurium (0.15–0.2 mg/kg), to achieve adequate muscle relaxation for endotracheal intubation. Maintenance included desflurane (end-tidal concentration 4–6%), cisatracurium (0.04–0.05 mg/kg), and opioids (fentanyl 1–2 µg/kg or morphine 0.1 mg/kg). Types of anesthetic medication and dosage were recorded. Blood transfusion and volume of intravenous fluid administration were recorded. After completion of surgery, the patients were extubated and transferred to the post-anesthesia care unit (PACU).

Decompressive laminectomy with spinal fusion was performed in the prone position. The surgical treatment involved the total excision of the lumbar spine lamina to perform a lumbar decompression. The posterolateral fusion with an autologous bone graft was performed in all patients, with or without using instruments. Pedicle screws and rods were applied to the spinal column in patients who had fusion with instrumentation [19,20]. The surgeons, board-qualified orthopedists, had over five years of experience carrying out decompressive laminectomy with spinal fusion. It was the decision of the surgeon as to whether or not to use an instrument during spinal fusion. Patients who reported moderate-to-severe pain were administered fentanyl 1 µg/kg in the PACU, with pain reassessed after 30 min; repeat dosing was permitted if necessary. Following reassessment, patients whose NRS pain score indicated mild pain were transferred back to the ward. Patients were categorized into either the parecoxib or control group based on their analgesic medication.

### 2.3. Parecoxib Group

An intravenous parecoxib 40 mg was administered at PACU if patients did not have any postoperative bleeding or any contraindications of parecoxib by surgeons. Then, parecoxib was subsequently given every 12 h for 48 h. The patients who reported moderate-to-severe pain additionally received intravenous morphine at 0.1 mg/kg, administered every 4–6 h as needed.

### 2.4. Control Group

The opioid group (control) included patients who received intravenous morphine 0.1 mg/kg and intravenous meperidine 1 mg/kg. These opioids were administered every 4–6 h as needed for moderate-to-severe pain.

Patients in both groups who reported mild pain would be given an oral paracetamol dose of 500 mg every 6 h. Opioid consumption during the first 96 h post-surgery was converted into milligrams of morphine equivalent (MME). The drainage tube was removed approximately on postoperative day 3, when the blood drainage volume was either 30 mL within 8 h or 100 mL within 24 h. Patients were categorized into the parecoxib group, whereas those who did not receive parecoxib were assigned to the control group.

### 2.5. Hospital Ambulation Protocol

Patients who experienced only mild pain during the first 24 h after surgery began bedside ambulation approximately 2 h postoperatively. This ambulation, supervised by staff nurses or physiotherapists, followed a structured stepwise approach that began with practicing the log-rolling technique, continued with maintaining proper spinal alignment using lumbosacral support, and gradually advanced to sitting up. The final step was ambulating out of bed, guiding patients step by step toward the ability to walk any distance with or without assistance [21,22].

The time to first ambulation was recorded. Early ambulation was defined as walking within 24 h after surgery, whereas walking beyond 24 h was considered late ambulation. Patients were allowed to begin ambulation as early as 8 h after surgery, provided their clinical condition was stable [23]. Early ambulation was undertaken according to the surgeon’s approval. The ambulation was recorded only when the patient was able to get out of bed and begin walking. The time of first ambulation out of bed in the postoperative period was recorded.

### 2.6. Outcomes

The primary outcome was the incidence of patients achieving early ambulation, defined as the ability to begin walking within 24 h after surgery. The secondary outcomes were the time to early ambulation, the amount of postoperative opioid consumption, LOS, the volume of blood drainage, and any side effects related to parecoxib and opioids.

### 2.7. Propensity Score Matching (PSM) Methods and Statistical Analyses

STATA version 16 (StataCorp LP, College Station, TX, USA) was utilized for data analysis. A two-sided *p*-value of 0.05 or lower was accepted as significant for all statistical analyses. Categorical data were presented as frequencies and percentages. Continuous variables were presented as mean and standard deviation or median and interquartile ranges as appropriate. To measure the average treatment effect of parecoxib on the ability of early ambulation after spine surgery, PSM has been employed. The matching was based on the following variables: age, gender, body mass index (BMI), significant underlying diseases (e.g., CAD, COPD, diabetes, CKD), type of spinal fusion, number of fusion levels, duration of the operation, amount of intraoperative opioid used, and postoperative opioid consumption at PAUC.

The matching ratio was 1:1 between patients who received opioids alone (control group) or the parecoxib group. PSM was chosen to enhance comparability between groups and to balance the distribution of confounding variables. The matching process aimed to balance the chosen covariates across treatment groups based on the calculated propensity scores. Histograms of propensity score distributions for both groups were plotted to illustrate the balance of scores before and after matching [24,25,26]. Logistic regression was used for the model construction. Each PSM cohort was compared by independent statistical techniques. A standardized difference (STD) of equal to or less than 10% was used as a threshold for demonstrating covariate balance. The sample size was calculated from the pilot review of 36 patients. The incidence of early ambulation was found to be 83% in the parecoxib group and 61% in the control group. The sample size calculation indicated that 65 patients per group was adequate to detect a 20% difference in the probability of early ambulation (power 80%, α = 0.05).

An exact probability test was used to compare categorical variables and differences between the two groups, and the independent two-sample Student’s *t*-test was applied to compare continuous data. A risk ratio (RR) was calculated to quantify the risk of late ambulation associated with parecoxib use, as well as the incidence of moderate-to-severe pain scores during the 96 h postoperative period. The number needed to treat (NNT) was calculated as the inverse of the risk difference and 95% confidence interval (CI).

Bonferroni correction was applied by dividing the conventional significance threshold. Pairwise comparisons between groups were performed using Fisher’s exact test, with the Bonferroni correction applied to adjust the significance threshold for multiple testing.

## 3. Results

**Figure 1:**
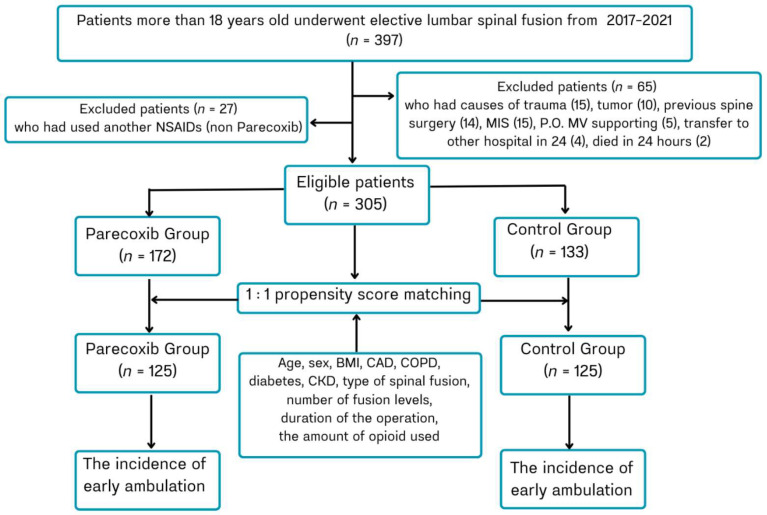
Study Flow Chart.

Between 2017 and 2021, a total of 397 patients underwent lumbar spine surgery. Ninety-two patients were excluded for various reasons, as illustrated in Figure 1. Among the remaining 305 patients, 172 received parecoxib, while 133 received opioids and their derivatives (control group).

Table 1 shows the patient characteristics before and after PSM. Before matching, the two groups differed significantly in terms of baseline characteristics and operative data, as demonstrated by the absolute value of STD over 10%. After the 1:1 PSM, two groups of 125 resulted. The patient characteristics and operative data were comparable between the groups. The STD was less than 10%.

**Figure 2:**
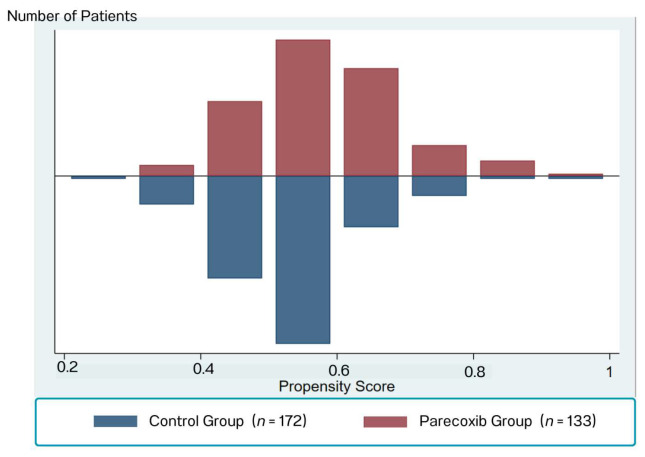
The Mean Propensity Scores in Each Group Before PSM.

**Figure 3:**
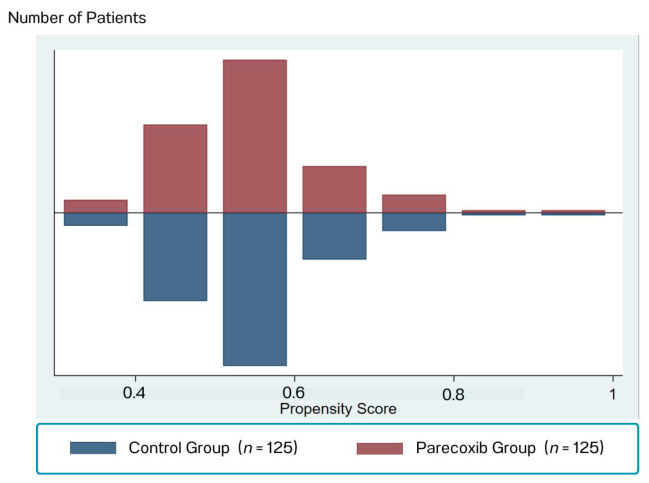
The Distribution of the Propensity Scores Between the Two Group After PSM.

The mean propensity scores in each group were significantly different before matching (0.59 ± 0.10 vs. 0.52 ± 0.20, *p* < 0.001), as illustrated in Figure 2. After 1:1 matching for the propensity score (by propensity score deciles), there were 125 patients included in each treatment group. The distribution of the propensity scores across the two groups was acceptable, as illustrated in Figure 3.

**Figure 4:**
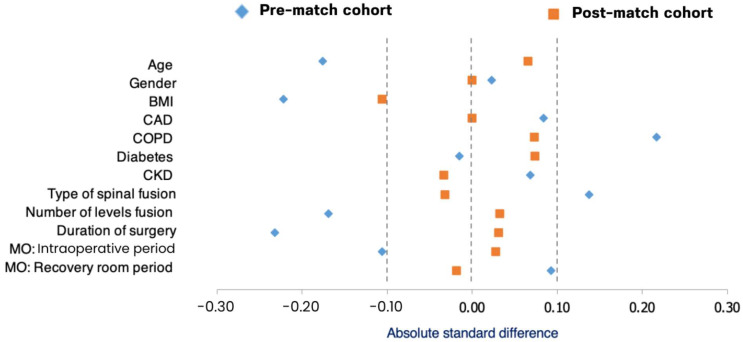
Standardized Mean Differences Before and After Propensity Score Matching. The diamond symbols and squares represent the standardized differences for the pre-matched cohort, while the squares show the differences for the post-matched cohort.

Figure 4 shows the distribution of STD for each covariate in the control and parecoxib groups before and after PSM. Before matching, several covariates exhibited notable imbalance between the two groups. After matching, all covariates showed substantially reduced STD, with all values falling within ±0.1, indicating adequate balance according to the predefined threshold. This demonstrates that the PSM procedure effectively balanced baseline characteristics between the parecoxib and control groups, thereby minimizing potential confounding effects. Achieving this covariate balance strengthens the validity of subsequent comparisons of postoperative outcomes between the two groups.

The incidence of early ambulation in the parecoxib group and the control group was 56% and 40%, respectively. Parecoxib significantly enhanced the probability of early ambulation by 39% (RR = 1.39, CI 1.06 to 1.84, *p* = 0.020). The number needed to treat (NNT) was calculated to be 6, indicating that six patients would need to receive parecoxib to prevent one case of late ambulation. Patients in the parecoxib group achieved early ambulation significantly faster (22. 7 vs. 33. 1 h, *p* < 0. 001), required significantly less opioid consumption over 96 h after lumbar spine fusion (9.6 vs. 13.2 MME, *p* < 0.001) and had significantly shorter length of hospital stay (6.1 vs. 9.6 days, *p* < 0.001) compared to those of the control group (Table 2).

**Figure 5:**
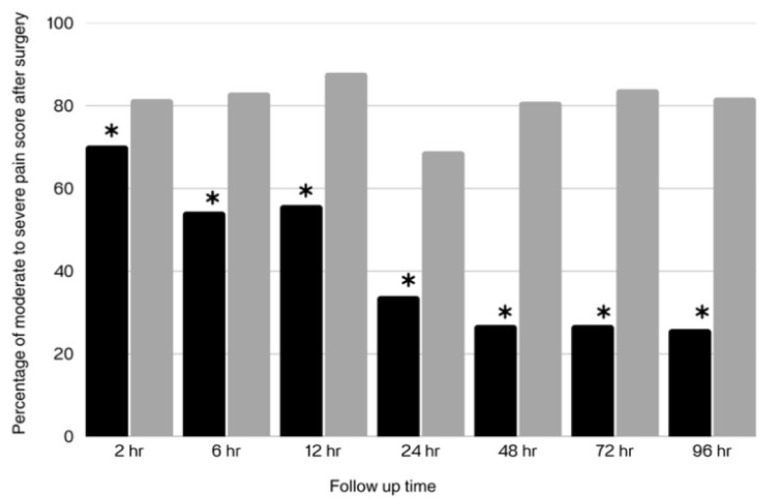
Percentage of Patients with Moderate-to-Severe Pain between the Parecoxib Group (black bars) and the Control Group (gray bars) Within 96 Hours after Surgery (Bonferroni correction, * *p* = 0.007).

Patients in the parecoxib group experienced significantly (*p* = 0.007) lower pain intensity than the patients in the control group, at all time points of pain assessment (Figure 5), and received 3.4 mg less opioids during the initial 96 h following surgery. There was no significant difference in the volume of blood drainage between the parecoxib group (121.8 ± 55.8 mL) and the control group (124.2 ± 54.8 mL; *p* = 0.739).

Adverse events included GI-related events (one in each group), acute kidney injury (one in the parecoxib group), and a thromboembolic event (one in the control group). No hypersensitivity or cardiovascular events were observed. Overall, these adverse events were infrequent and showed no significant difference between groups (all *p* > 0.05).

## 4. Discussion

This study found that patients receiving parecoxib after lumbar spine surgery had a higher incidence of early ambulation, achieved a shorter time to ambulation, required less opioid analgesia, reported a lower percentage of patients with moderate-to-severe NRS pain scores over the 96 h postoperative period, and had shorter hospital stays compared to controls. Early ambulation is an intervention preventing postoperative complications after spine surgery. It facilitates venous return, thereby lowering the risk of thrombus formation and clot dislodgement [10]. Although the overall incidence is relatively low, these complications are potentially life-threatening. Thus, encouraging early ambulation not only reduces the risk of PE and DVT but also contributes to improved recovery, better patient outcomes, and increased patient satisfaction [6,7,10].

Supporting this observation, a randomized controlled study by Chiu et al. compared 40 mg IV parecoxib (every 12 h for 72 h) with patient-controlled analgesia (PCA) morphine in lumbar spinal fusion patients and found that parecoxib provided superior analgesia and significantly improved postoperative ambulation, as reflected by a significantly greater improvements in Barthel Index (BI) scores. BI scores increased significantly from baseline in both groups; however, improvements were greater in the parecoxib group at 48 hours (+4.36 points, *p* = 0.04) and 72 hours (+6.26 points, *p* < 0.01) postoperatively, indicating faster recovery of daily activities [12]. These findings highlight that parecoxib accelerates postoperative functional recovery. The improvements in transfers, standing, and walking represent key elements of early ambulation, suggesting that parecoxib may facilitate earlier ambulation, although direct time-to-ambulation outcomes were not assessed in that study. Our findings further show that parecoxib accelerated ambulation after lumbar spine surgery and that this earlier ambulation was associated with a shorter hospital stay.

Several studies have compared different analgesic techniques and reported outcomes related to ambulation. For example, Zhang et al. conducted a randomized clinical trial in patients undergoing posterior lumbar decompression surgery and demonstrated that the time to first ambulation was significantly shorter in the erector spinae plane block (ESPB) group (median 26.0 h [IQR 22.0–30.0]) compared with controls (median 32.0 h [IQR 27.0–38.0] *p* = 0.006), suggesting that ESPB facilitates earlier mobilization. The control group underwent the same surgical and anesthetic management but did not receive a preoperative bilateral ultrasound-guided ESPB, representing standard postoperative analgesic care [27]. However, their study was limited to decompression procedures, whereas our investigation involved spinal fusion surgery. Fusion procedures generally require more extensive tissue dissection, longer operative times, and greater postoperative pain, all of which present additional challenges to early ambulation despite optimized analgesia.

The study has reported no significant effect of analgesic regimens on postoperative ambulation. Li et al. investigated adolescents undergoing posterior spinal fusion for idiopathic scoliosis and compared intrathecal morphine (ITM) alone with intrathecal morphine plus gabapentin. They found no significant difference in time to first ambulation between the two groups (20.7 ± 8.1 h vs. 19.3 ± 5.5 h, *p* = 0.475) [28]. In contrast, our study specifically demonstrated that parecoxib had a significant impact on both the incidence and timing of ambulation, adding new evidence on this clinically important outcome that has been rarely examined in previous analgesic trials for spine surgery.

In a recent systematic review and meta-analysis of 27 randomized controlled trials (*n* = 2840), the efficacy and safety of intravenous parecoxib during the perioperative period for postoperative pain relief after orthopedic surgery, including lumbar spine surgery, total knee arthroplasty (TKA), total hip arthroplasty (THA), and other common orthopedic operations, were evaluated. Among the 27 trials, five involved lumbar spine surgery; of these, three studies demonstrated that parecoxib significantly reduced postoperative morphine consumption within 24 h compared to the control. One non-randomized observational study in patients undergoing lumbar spinal fusion administered parecoxib as a 40 mg intravenous bolus perioperatively, followed by 40 mg every 12 h for 72 h. Patients who received parecoxib had a 39% reduction in total morphine consumption over 48 h, and a 30% reduction in pain at rest (NNT = 3.1, 95% CI: 2.0–4.6) [15]. Consistently, our findings suggest that parecoxib enhances postoperative pain control and reduces opioid use, potentially lowering opioid-related side effects. However, patients undergoing spine surgery often experience substantial pain when moving or attempting to ambulate; therefore, multimodal analgesia strategies—including the use of parecoxib as part of an opioid-sparing regimen—are recommended to facilitate early ambulation [12,13,14,15]. Previous studies have demonstrated that parecoxib is effective in reducing postoperative pain following lumbar spine surgery and in decreasing the need for opioid analgesics [2,3,4,13,15].

The serious adverse events associated with parecoxib—including gastrointestinal, renal, cardiovascular, and hypersensitivity reactions—have been reported only rarely in perioperative use [15,29]. These findings support the short-term use of parecoxib as a safe component of multimodal analgesia following spine surgery, provided that patients are carefully monitored for known class-related risks. According to Schug SA, the incidence of postoperative nausea and vomiting (PONV), hypersensitivity reactions, and AKI did not differ between patients treated with parecoxib and those treated with opioids. Moreover, no gastrointestinal-related adverse events or thromboembolic complications were reported in either treatment group [29,30]. In our study, the overall safety profile of parecoxib was favorable, with no hypersensitivity or cardiovascular complications reported. In addition, the opioid-sparing effect of parecoxib may help reduce common opioid-related adverse effects, such as nausea, vomiting, pruritus, sedation, and respiratory depression [31]. These findings support the implementation of parecoxib for postoperative pain control within pathways such as ERAS protocols for lumbar spine fusion, with demonstrated benefits in promoting early ambulation, reducing opioid consumption, and shortening hospitalization.

### 4.1. Strengths

This study employed the statistical approach of PSM to balance the distribution of confounding variables between groups. PSM is particularly indicated in observational or non-randomized studies, where treatment allocation is not randomized and baseline characteristics may differ between groups. It can be applied when detailed individual-level data on potential confounders (e.g., demographic, clinical, or perioperative variables) are available. By creating matched sets of patients with similar propensity scores, PSM reduces selection bias and approximates the conditions of a randomized controlled trial, thereby enhancing the validity of the estimated treatment effect.

We chose PSM instead of inverse probability of treatment weighting (IPTW) to enhance the comparability between treatment groups and to minimize model dependence. PSM allows for direct pairing of patients with similar baseline covariates and restricts the analysis to subjects within the region of common support. In contrast, IPTW can produce extreme or unstable weights, especially when the overlap between treated and control groups is limited, which may inflate the variance and bias of estimated treatment effects [23,24,25].

Therefore, this study provides evidence supporting the analgesic efficacy of parecoxib in facilitating early ambulation following lumbar spine surgery. The follow-up period was 96 h after surgery.

### 4.2. Limitations

The first limitation is that this is a retrospective study, in which the data available for analysis may not be fully complete. In particular, preoperative pain history—whether chronic or acute—was not consistently documented, which could have influenced postoperative pain levels. Consequently, this variable was considered as a potential source of confounding by indication during the PSM procedure. Secondly, although the parecoxib group exhibited no serious adverse effects, the sample size was not specifically calculated based on the expected incidence of adverse effects. Third, this study was conducted at a single tertiary care center, which may limit the generalizability of the findings. This approach may limit the generalizability of the results, as patient demographics, surgical practices, and postoperative care may vary between healthcare facilities. A future randomized controlled trial will enhance the reliability of the outcomes.

## 5. Conclusions

This study demonstrated that intravenous parecoxib significantly increased the incidence of early ambulation and shortened the time to initiate walking after elective lumbar spine fusion, compared with conventional opioid analgesia, without causing serious adverse events. Parecoxib also significantly reduced the amount of postoperative opioid consumption within 96 h and shortened the LOS. This intervention may be an effective component of multimodal analgesia within an ERAS protocol for lumbar spine surgery. Further randomized controlled trials with larger sample sizes are warranted to validate these findings and to assess the broader impact of parecoxib on functional recovery and long-term postoperative outcomes.

## Figures and Tables

**Table 1:** Patient Characteristics and Operative Data Before and After Propensity Score Matching (PSM).

	Before PSM			After PSM		
Characteristics and Operative Data	Parecoxib Group (*n* = 172) Mean ± SD	Control Group (*n* = 133) Mean ± SD	STD %	Parecoxib Group (*n* = 125) Mean ± SD	Control Group (*n* = 125) Mean ± SD	STD %
Age (Years)	59.9 ± 8.3	60.1 ± 8.6	−17.55	59.4 ± 8.6	59.9 ± 8.3	6.58
Gender, *n* (%)						
: Male	124 (72.1)	85 (63.9)	2.28	84 (67.2)	84 (67.2)	0.00
: Female	48 (27.9)	48 (36.1)		41 (32.8)	41 (32.8)	
BMI (kg/m^2^)	25.6 ± 4.2	24.7 ± 4.1	−22.17	25.3 ± 4.3	24.8 ± 4.2	−10.57
Comorbidity, *n* (%)						
: CAD	2 (1.2)	3 (2.3)	8.41	2 (1.6)	2 (1.6)	0.00
: COPD	7 (4.1)	1 (0.8)	21.69	2 (1.6)	1 (0.8)	7.32
: Diabetes	39 (22.7)	31 (23.3)	−1.50	39 (26.4)	29 (23.2)	7.39
: CKD	10 (5.8)	10 (7.5)	6.82	8 (6.4)	7 (5.6)	−3.36
Type of fusion, *n* (%)						
: BG and instrumental fusion	101 (58.7)	69 (51.9)	13.75	65 (52.0)	67 (53.6)	−3.19
: BG fusion alone	71 (41.3)	64 (48.1)		60 (48.0)	58 (46.4)	
Fusion level, *n* (%)						
: 1–2	79 (45.9)	50 (37.6)	−16.91	48 (45.9)	50 (59.0)	3.27
: ≥3	93 (54.1)	83 (62.4)		77 (54.1)	75 (39.0)	
Duration of surgery (minutes)	329.8 ± 166.6	288.5 ± 188.9	−23.19	291.5 ± 149.6	296.9 ± 191.6	3.14
Amount of opioid used (MME)						
: Intra-operative period	14.3 ± 4.0	13.9 ± 4.1	−10.60	13.8 ± 4.0	13.9 ± 4.1	2.79
: PACU period	3.2 ± 2.6	3.4 ± 2.7	9.36	3.3 ± 2.7	3.3 ± 2.6	−1.84
Propensity score	0.59 ± 0.1	0.52 ± 0.1	−47.01	0.55 ± 0.1	0.54 ± 0.1	−4.35

Frequency and percentage: *p*-values (Fisher’s exact test), Mean ± SD: *p*-values (*t*-test analysis). STD (standardized difference), SD (standard deviation), BMI (body mass index), CAD (coronary artery disease), COPD (chronic obstructive pulmonary disease), CKD (chronic kidney disease), BG (bone graft), MME (milligrams of IV morphine equivalent).

**Table 2:** Comparison of Postoperative Outcomes Between the Parecoxib Group and Control Group.

Post-Operative Outcomes	Parecoxib Group (*n* = 125) Mean ± SD	Control Group (*n* = 125) Mean ± SD	Mean Difference	95% CI	*p*-Value
Time to early ambulation (hours)	22.7 ± 4.3	33.1 ± 12.8	−10.448	−12.836, −8.060	<0.001 **
Total opioid requirement (MME)	9.6 ± 6.1	13.2 ± 6.6	−3.632	−5.213, −2.051	<0.001 **
Volume of blood drainage (mL)	123.8 ± 55.7	122.1 ± 53.7	−1.720	−11.919, 15.359	0.804
LOS (days)	6.1 ± 2.3	9.6 ± 2.9	−3.552	−4.201, −2.903	<0.001 **

Frequency and percentage: *p*-values (Fisher’s exact test), Mean ± SD: *p*-values (*t*-test analysis). Mean Difference: ** *p*-values (*t*-test analysis) <0.05 significance, CI (confidence interval), SD (standard deviation), MME (milligram of IV morphine equivalents), mL (milliliter), LOS (length of stay).

## Data Availability

Data sets generated or analyzed in this study are accessible upon reasonable request from the corresponding author.

## Abbreviations

IV	Intravenous
PSM	Propensity score matching
DVT	Deep Vein Thrombosis
PE	Pulmonary Embolism
ERAS	Enhanced Recovery After Surgery
NSAIDs	Non-Steroidal Anti-Inflammatory Drugs
COX-2	Cyclooxygenase 2
PROSPECT	Procedure-Specific postoperative pain management
ICD-10	International Classification of Diseases 10
IRB	Institutional Review Board
BMI	Body Mass Index
CAD	Coronary Artery Disease
COPD	Chronic Obstructive Pulmonary Disease
CKD	Chronic Kidney Disease
NRS	Numerical Rating Scale
GI	Gastrointestinal
AKI	Acute Kidney Injury
KDIGO	Kidney Disease: Improving Global Outcomes
LOS	Length of Stay
ECG	Electrocardiogram
ETCO2	End-Tidal Carbon Dioxide
BG	Bone Graft
PACU	Post-Anesthesia Care Unit
MME	Milligrams of IV Morphine Equivalent
STD	Standardized Difference
RR	Risk Ratio
NNT	Number Needed to Treat
mL	milliliter
CI	Confidence Interval
SD	Standard Deviation
PCA	Patient-Controlled Analgesia
BI	Barthel Index
ESPB	Erector Spinae Plane Block
IQR	Interquartile Range
ITM	Intrathecal Morphine
TKA	Total Knee Arthroplasty
THA	Total Hip Arthroplasty
PONV	Postoperative Nausea and Vomiting
IPTW	Inverse Probability of Treatment Weighting

## References

[1] Bajwa S.J., Haldar R. (2015). Pain management following spinal surgeries: An appraisal of the available options. J. Craniovertebr. Junction Spine.

[2] Peene L., Le Cacheux P., Sauter A.R., Joshi G.P., Beloeil H. (2021). Pain management after laminectomy: A systematic review and procedure-specific post-operative pain management (prospect) recommendations. Eur. Spine J..

[3] Waelkens P., Alsabbagh E., Sauter A., Joshi G.P., Beloeil H. (2021). Pain management after complex spine surgery: A systematic review and procedure-specific postoperative pain management recommendations. Eur. J. Anaesthesiol..

[4] Mathiesen O., Dahl B., Thomsen B.A., Kitter B., Sonne N., Dahl J.B., Kehlet H. (2013). A comprehensive multimodal pain treatment reduces opioid consumption after multilevel spine surgery. Eur. Spine J..

[5] Gerbershagen H.J., Aduckathil S., van Wijck A.J., Peelen L.M., Kalkman C.J., Meissner W. (2013). Pain intensity on the first day after surgery: A prospective cohort study comparing 179 surgical procedures. Anesthesiology.

[6] Burgess L.C., Wainwright T.W. (2019). What Is the Evidence for Early Mobilisation in Elective Spine Surgery? A Narrative Review. Healthcare.

[7] Huang J., Shi Z., Duan F.F., Fan M.X., Yan S., Wei Y., Han B., Lu X., Tian W. (2021). Benefits of Early Ambulation in Elderly Patients Undergoing Lumbar Decompression and Fusion Surgery: A Prospective Cohort Study. Orthop. Surg..

[8] Inturrisi C.E. (2002). Clinical pharmacology of opioids for pain. Clin. J. Pain.

[9] Cheung C.K., Adeola J.O., Beutler S.S., Urman R.D. (2022). Postoperative Pain Management in Enhanced Recovery Pathways. J. Pain Res..

[10] Debono B., Wainwright T.W., Wang M.Y., Sigmundsson F.G., Yang M.M.H., Smid-Nanninga H., Bonnal A., Le Huec J.-C., Fawcett W.J., Ljungqvist O. (2021). Consensus statement for perioperative care in lumbar spinal fusion: Enhanced Recovery After Surgery (ERAS^®^) Society recommendations. Spine. J..

[11] Derry S., Moore R.A. (2013). Single dose oral celecoxib for acute postoperative pain in adults. Cochrane. Database. Syst. Rev..

[12] Chiu S.C., Livneh H., Chen J.C., Chang C.M., Hsu H., Chiang T.I., Tsai T.-Y. (2022). Parecoxib Reduced Postsurgical Pain and Facilitated Movement More Than Patient Controlled Analgesia. Front. Surg..

[13] Moonla R., Threetipayarak A., Panpaisarn C., Pattayaruk N., Kaewkam U., Jumpalee N., Panwilai J. (2018). Comparison of Preoperative and Postoperative Parecoxib Administration for Pain Control Following Major Spine Surgery. Asian. Spine. J..

[14] Zhang Z., Xu H., Zhang Y., Li W., Yang Y., Han T., Wei Z., Xu X., Gao J. (2017). Nonsteroidal anti- inflammatory drugs for postoperative pain control after lumbar spine surgery: A meta- analysis of randomized controlled trials. J. Clin. Anesth..

[15] Li X., Zhou P., Li Z., Tang H., Zhai S. (2022). Intravenous Parecoxib for Pain Relief after Orthopedic Surgery: A Systematic Review and Meta-analysis. Pain Ther..

[16] Liu Y., Xiao S., Ya H., Lv X., Hou A., Ma Y., Jiang Y., Duan C., Mi W., CAPOPS Group (2023). Postoperative pain-related outcomes and perioperative pain management in China: A population-based study. Lancet Reg. Health West. Pac..

[17] Pipanmekaporn T., Punjasawadwong Y., Charuluxananan S., Lapisatepun W., Bunburaphong P., Boonsri S., Tantraworasin A., Bunchungmongkol N. (2017). The effectiveness of intravenous parecoxib on the incidence of ipsilateral shoulder pain after thoracotomy: A randomized, double-blind, placebo-controlled trial. J. Cardiothorac. Vasc. Anesth..

[18] Kellum J.A., Lameire N., KDIGO AKI Guideline Work Group (2013). Diagnosis, Evaluation, and Management of Acute Kidney Injury: A KDIGO Summary (Part 1). Crit. Care.

[19] Ghogawala Z., Dziura J., Butler W.E., Dai F., Terrin N., Magge S.N., Coumans J.-V.C., Harrington J.F., Amin-Hanjani S., Schwartz J.S. (2016). Laminectomy plus Fusion versus Laminectomy Alone for Lumbar Spondylolisthesis. N. Engl. J. Med..

[20] Lee S.C., Chen J.F., Wu C.T., Lee S.T. (2009). In situ local autograft for instrumented lower lumbar or lumbosacral posterolateral fusion. J. Clin. Neurosci..

[21] Haddas R., Remis A., Barzilay Y., Puvanesarajah V., Keller J., Clifford B.M., Lantz J.M., Mayer J.M. (2025). Therapeutic exercise following lumbar spine surgery: A narrative review. N. Am. Spine Soc. J..

[22] Sakaguchi T., Gunjotikar S., Tanaka M., Komatsubara T., Latka K., Ekade S.J., Prabhu S.P., Takamatsu K., Yasuda Y., Nakagawa M. (2024). Evaluation and Rehabilitation after Adult Lumbar Spine Surgery. J. Clin. Med..

[23] Lim S., Bazydlo M., Macki M., Haider S., Hamilton T., Hunt R., Chaker A., Kantak P., Schultz L., Nerenz D. (2022). Validation of the Benefits of Ambulation Within 8 Hours of Elective Cervical and Lumbar Surgery: A Michigan Spine Surgery Improvement Collaborative Study. Neurosurgery..

[24] Austin P.C. (2011). An Introduction to Propensity Score Methods for Reducing the Effects of Confounding in Observational Studies. Multivariate Behav. Res..

[25] Stuart E.A. (2010). Matching methods for causal inference: A review and a look Forward. Stat. Sci..

[26] Austin P.C. (2013). A comparison of 12 algorithms for matching on the propensity score. Stat. Med..

[27] Zhang T., Zhang J., Qu Z., Hua Z., Sun Y. (2025). A randomized clinical trial of erector spinae plane block and chronic pain after posterior lumbar surgery. Sci. Rep..

[28] Li Y., Swallow J., Robbins C., Caird M.S., Leis A., Hong R.A. (2021). Gabapentin and intrathecal morphine combination therapy results in decreased oral narcotic use and more consistent pain scores after posterior spinal fusion for adolescent idiopathic scoliosis. J. Orthop. Surg. Res..

[29] Schug S.A., Parsons B., Li C., Xia F. (2017). The safety profile of parecoxib for the treatment of postoperative pain: A pooled analysis of 28 randomized, double-blind, placebo-controlled clinical trials and a review of over 10 years of postauthorization data. J. Pain Res..

[30] Schug S.A. (2006). The Role of COX-2 Inhibitors in the Treatment of Postoperative Pain. J. Cardiovasc. Pharmacol..

[31] Jirarattanaphochai K., Thienthong S., Sriraj W., Jung S., Pulnitiporn A., Lertsinudom S., Foocharoen  T. (2008). Effect of parecoxib on postoperative pain after lumbar spine surgery: A bicenter, randomized, double-blinded, placebo-controlled trial. Spine.

